# Comparative evaluation of the antipyretic and analgesic efficacy and safety of a fast-release paracetamol 650 mg formulation vs. conventional paracetamol formulations in adults with acute febrile or febrile-pain conditions: a multicenter randomized controlled trial

**DOI:** 10.3389/fpain.2026.1965374

**Published:** 2026-09-30

**Authors:** Susobhan Mondal, S. K. Ashik Ikbal, Sumit Agrawal, Amar Raykantiwar, Avirath Acchammachary, Suhas Kalashetti, K. R. Shivaprasad, Ashok V B, Mukulesh Gupta, Vikas Verma, Monjori Mitra, Amitrajit Pal, Dattatray Pawar, Akhilesh Sharma

**Affiliations:** 1 Apollo Clinic, Salt Lake, Kolkata, India; 2 Olivia Nursing Home, Dum Dum, Kolkata, India; 3 Agrawal Hospital, Pune, India; 4 Atharva Diabetes and Weight Loss Center, Pune, India; 5 Silver Birch Multispecialty Hospital, Pune, India; 6 Shree Samarth Hospital, Pune, India; 7 Dooravani Diagnostics and Multispecialty Clinic, Bangalore, India; 8 Dr. Ashok's Clinic, Bangalore, India; 9 Udyan Healthcare Pvt Ltd., Lucknow, India; 10 Vardhman Hospital, Lucknow, India; 11 Department of Pediatrics, Institute of Child Health, Kolkata, West Bengal, India; 12 Alkem Laboratories Ltd., Mumbai, Maharashtra, India

**Keywords:** Acetaminophen, analgesic, antipyretic, fever, pain, paracetamol, rapid relief

## Abstract

**Aim:**

Paracetamol is widely used for management of fever/pain. With increasing emphasis on rapid symptom relief, this study compared the response following a single 650 mg oral dose of four commercially available tablets in acute febrile and febrile-pain conditions.

**Methods:**

This multicenter, open-label trial (CTRI/2025/12/098995) included adults (18–60 years) with oral temperature ≥38 °C (with/without pain) and symptom onset within ≤72 h. Consenting participants were randomized (1:1:1:1) to receive a single dose of the test fast release test paracetamol (A) and conventional paracetamol (B, C, D) drug. Oral body temperature(digital thermometer) and pain (numerical rating scale (NRS) were assessed at predefined intervals till 90 ± 2 min. Outcomes included magnitude and onset of antipyretic and analgesic effects, adverse events (AEs), and patient satisfaction.

**Results:**

Among 220 febrile participants (55% women; mean temperature 38.51 °C), 169 reported pain (mean NRS score 7.31). At 90 ± 2 min, fast release paracetamol demonstrated significantly greater reduction in temperature (1.54 °C), along with the fastest rate of decline (1.03 °C/hr), and the shortest restricted mean time to defervescence (84.27 min), resulting in majority of participants (60.0%) experiencing defervescence (temperature ≤37 °C). Pain reduction was also significantly greater with fast release test paracetamol (5.2 units; 3.4 units/hr), with onset of analgesia (≥30% reduction in NRS score) after a comparatively shorter median time (30 min; conventional, 60–90 min). By 90 ± 2 min, pain resolved (NRS score = 0) in 23.2% (A), 22.5% (B), 11.9% (C), and 6.8% (D) participants, with restricted mean times to resolution being 90.00, 89.25, 89.29, and 90.00 min, respectively. Overall, 82.2% participants reported treatment satisfaction, with likelihood highest for fast-release test paracetamol (A). Four participants reported unrelated, mild/moderate AEs.

**Conclusion:**

All evaluated paracetamol 650 mg formulations demonstrated antipyretic and analgesic activity within 90 min, while the fast-release formulation showed earlier and, for several assessed outcomes, greater reductions in temperature and pain. These findings may support consideration of formulation choice when rapid early symptomatic relief is desired.

**Clinical Trial Registration:**

CTRI/2025/12/098995.

## 1 Introduction

Fever and associated pain can adversely affect the quality of life, daily functioning, and productivity, particularly among working-age adults, highlighting the need for prompt and effective symptom management. Paracetamol/acetaminophen is one of the most commonly used oral antipyretics and analgesic agents worldwide, and many patients prefer oral medications as they are easy to consume, widely available, and usually more cost-effective than other formulations (1). The onset and consistency of action following oral paracetamol can be influenced by physiological factors such as gastric emptying and formulation characteristics, and differences in formulation type, for example, immediate-release compared with modified-or sustained-release, can alter pharmacokinetic profiles in healthy subjects (1–3).

Pharmacokinetic variability across different formulations and brands may affect both the speed and intensity of antipyretic and analgesic effects, with some studies noting variations in absorption rates and plasma concentrations that could translate into differences in onset of symptom relief (2, 3). Since early relief of symptoms is important for patient satisfaction and adherence, such differences warrant clinical consideration.

Safety also remains a key consideration, particularly with repeated dosing, because of known risks of hepatotoxicity at higher cumulative doses (4). Moreover, demonstrating bioequivalence between generic oral formulations does not always guarantee equivalent clinical performance, particularly for rapid symptom relief (2). Although bioequivalent formulations are generally expected to provide comparable therapeutic efficacy, differences in early symptomatic response may still be clinically relevant in acute febrile illnesses where rapid relief is desirable.

In routine clinical practice, paracetamol is frequently used as an over-the-counter medication for self-management of fever and associated pain. Despite its widespread use, comparative clinical data evaluating early antipyretic and analgesic responses among commonly prescribed oral paracetamol formulations under similar clinical conditions remain limited, particularly in Indian settings where the 650 mg dose is extensively used.

Although fast-acting paracetamol formulations have previously been evaluated, direct head-to-head clinical comparisons of multiple marketed 650 mg formulations under the same randomized clinical and early assessment conditions remain limited, particularly in Indian clinical settings. The the principal contribution of the present study is the comparative characterization of the early antipyretic and analgesic responses to four commercially available paracetamol 650 mg formulations rather than the evaluation of a novel pharmacological mechanism. Accordingly, this multicenter, randomized, controlled study compared their early antipyretic and analgesic effects, patient-reported treatment satisfaction, and short-term tolerability in adults with acute febrile or febrile-pain conditions.

## 2 Materials and methods

### 2.1 Study design

This was a prospective, open-label, multicenter, randomized, controlled trial conducted from December 2025 to January 2026 at ten sites across India, namely, Apollo clinic (Kolkata), Olivia Nursing Home (Kolkata), Agrawal Hospital (Pune), Atharva Diabetes and Weight Loss Center (Pune), Silver Birch Multispecialty Hospital (Pune), Shree Samarth Hospital (Pune), Dooravani Diagnostics and Multispecialty Clinic (Bangalore), Dr. Ashok's Clinic (Bangalore), Udyan Healthcare Pvt. Ltd. (Lucknow), and Vardhman Hospital (Lucknow).

### 2.2 Ethical considerations

This study was conducted in accordance with the Declaration of Helsinki, Good Clinical Practice (GCP) guidelines as per the International Council for Harmonization (ICH) of technical requirements for pharmaceuticals for human use, and Guidelines for Clinical Trials on Pharmaceutical Products in India as specified in the New Drugs and Clinical Trials Rules, 2019. The study was approved by the Institutional Ethics Committees at all sites and registered with the Clinical Trials Registry-India (CTRI/2025/12/098995). All participants provided written informed consent before enrolment.

### 2.3 Participants

Adult male or female patients aged between 18 and 60 years (both inclusive) were eligible to participate, provided they presented with a body temperature (oral) of ≥38 °C (with/without associated pain) and had symptom onset within 72 h preceding screening. Patients with known allergy/hypersensitivity/contraindications to paracetamol/acetaminophen, fever/pain caused due to chronic clinical conditions (e.g., osteoarthritis, tuberculosis, malignancy, etc.), known liver dysfunction, use of any antipyretic or analgesic within 12 h before screening, requiring hospitalization at baseline, or pregnancy or lactation were excluded.

### 2.4 Treatment

Eligible participants were randomly allocated using a computer-generated allocation sequence, in a 1:1:1:1 ratio, to receive a single dose of one of the following four oral paracetamol 650 mg tablets: (a) test medication Fast release test paracetamol (A) (SUMO-L™ Fast 650 tablet; Alkem Laboratories), and (b) paracetamol B (Dolo-650® tablet; Micro Labs Ltd.), (c) paracetamol C (Calpol® 650 tablet; Glaxo SmithKline Pharmaceuticals Ltd.), (d) paracetamol D (unbranded/generic paracetamol tablets IP 650 mg). Randomization was performed centrally, with treatment assignment implemented sequentially at each site. Following baseline assessment of oral temperature and NRS pain score, participants received the assigned single dose of paracetamol and were monitored in person for 90 min. No protocol-mandated fasting or fed condition was specified for study-drug administration. Pain intensity was self-reported by participants using an 11-point numerical rating scale (NRS) ranging from 0 (“no pain”) to 10 (“worst imaginable pain”). Participants recorded their pain score at baseline and at 10 ± 2, 15 ± 2, 20 ± 2, 30 ± 2, 45 ± 2, 60 ± 2, and 90 ± 2 min after dosing. Study personnel at all participating sites were trained before study initiation on standardized protocol-specific procedures, including while taking oral temperature measurement, administration of the NRS, adherence to the predefined assessment schedule, and recording of study data. Temperature and pain score were recorded at 10, 15, 20, 30, 45, 60, and 90 min, with a permissible window period of ±2 for each timepoint. Adverse events (AEs) and serious AEs (SAEs) were monitored throughout the duration. Treatment satisfaction was assessed once at 90 ± 2 min after dosing using a 5-point Likert scale: 1 = very unsatisfied, 2 = unsatisfied, 3 = neutral, 4 = satisfied, and 5 = very satisfied. For the responder analysis, participants selecting a score of 4 (“satisfied”) or 5 (“very satisfied”) were classified as satisfied.

### 2.5 Endpoints

The following were the primary endpoints: (i) change in body temperature (oral) from baseline to 10 ± 2, 15 ± 2, 20 ± 2, 30 ± 2, 45 ± 2, 60 ± 2, and 90 ± 2 min; (ii) rate of fall of baseline body temperature (defined as decrease in temperature per minute); and (iii) time to ≥1 °C reduction in baseline body temperature (oral).

Secondary endpoints included the following: (i) change in pain score from baseline to 10 ± 2, 15 ± 2, 20 ± 2, 30 ± 2, 45 ± 2, 60 ± 2, and 90 ± 2 min based on the NRS scale; (ii) rate of decrease of baseline pain score (defined as decrease in pain score per minute); (iii) time to ≥30% reduction in baseline NRS score; (iv) time to fever resolution (defined as body temperature ≤37 °C); (v) proportion of participants achieving complete fever resolution at 90 ± 2 min; (vi) proportion of patients with ≥30% reduction in baseline NRS score at 10 ± 2, 15 ± 2, 20 ± 2, 30 ± 2, 45 ± 2, 60 ± 2, and 90 ± 2 min; (vii) time to pain resolution (defined as NRS score of 0); (viii) proportion of patients achieving complete pain resolution (defined as attainment of NRS score of 0) at 90 ± 2 min; (ix) proportion of patients with treatment satisfaction for resolution of febrile or febrile-pain conditions (based on a 5-point Likert scale: 1 = very unsatisfied; 2 = unsatisfied; 3 = neutral; 4 = satisfied; 5 = very satisfied) at 90 ± 2 min after dosing; (x) proportion of participants with AEs and SAEs.

### 2.6 Sample size

In order to detect a clinically relevant decrease of 1 °C in baseline body temperature with a conservative estimate of 1.5 °C as the assumed variability of temperature reduction consistent with a prior randomized trial (5), the sample size for this study was computed as 220 in total and 55 per group, considering power of 90%, alpha 0.05, and 10% attrition rate.

### 2.7 Statistical methods

All data analyses were based on the ICH E9 document 'Statistical Principles for Clinical Trials' and done using R 4.5.2. Continuous variables were reported as mean ± standard deviation (SD), median, minimum, maximum, or 95% confidence interval (CI), as appropriate. Continuous variables were analyzed using repeated measures ANOVA to assess both within-group and between-group changes over time, if the data was normally distributed. If the data was not normally distributed, Wilcoxon signed rank test/Mann Whitney test was used. Frequency and proportions were reported for categorical variables. Chi-square/Fisher's exact test was used to compare independent proportions/percentages and McNemar test for paired comparisons. Time-to-event data were analyzed using Kaplan–Meier estimator to find the median along with 95% CI and number of events. In cases where the median time could not be calculated, cumulative event probabilities were reported at each time point. Cox regression was used to calculate hazard ratio (HR) and binary logistic regression was used to calculate odds ratio (OR), when appropriate. Generalized estimating equation (GEE) model was used to analyze the binary responses of achieving a particular event at each timepoint, adjusting within-subject correlation due to repeated measurements. Estimated Marginal Means (EMMs) were used to derive odds of response with 95% CIs between treatment groups at each time point, with the test group as the reference. All statistical tests were two-sided; *p*-value < 0.05 was considered statistically significant.

## 3 Results

### 3.1 Participant disposition

This trial is reported in accordance with the CONSORT 2010 guidelines. A total of 220 participants were screened and enrolled; 55 participants were randomized to each treatment group, all of whom completed the study. There were no screen failures or dropouts (Figure 1).

**Figure 1:**
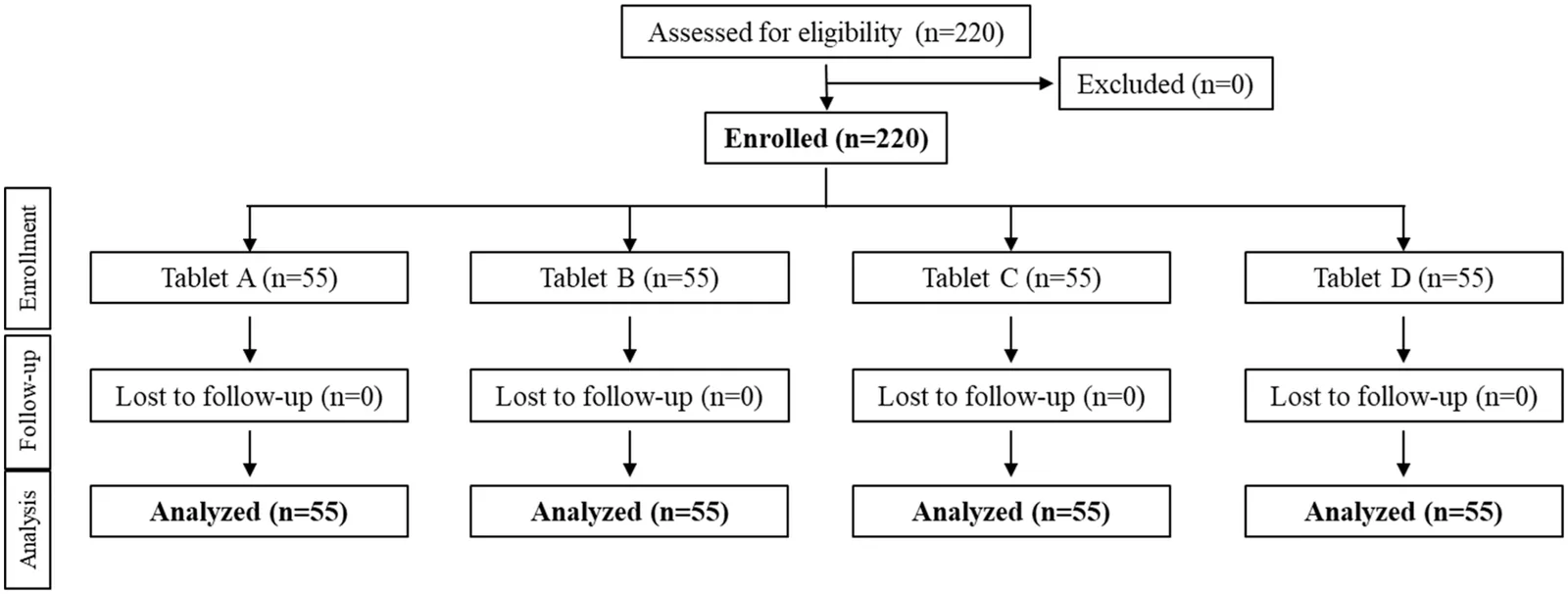
CONSORT flow ndiagram.

### 3.2 Baseline characteristics of participants

Among enrolled participants, 55.0% were women and 45.0% men. Overall, the mean age was 37 years and the mean weight was 67.71 kg. All participants were febrile at baseline and 76.8% had associated pain. Overall, the mean body temperature at baseline was 38.51 °C (range: 38.0–40.0 °C) and the mean NRS pain score was 7.31 (range: 3–9). Baseline characteristics were comparable across the treatment groups (*p* > 0.05) (Table 1).

**Table 1:** Demographic and clinical characteristics of participants at baseline.

Characteristic	Total (*N* = 220)	Fast-release test paracetamol (A) (*n* = 55)	Paracetamol (B) (*n* = 55)	Paracetamol (C) (*n* = 55)	Paracetamol (D) (*n* = 55)	*p*-value
Age (years)^b^, Mean ± SD	37 ± 9	38. ± 9	36 ± 8	37 ± 9	36 ± 9	0.729
Weight (kg)^b^, Mean ± SD	67.71 ± 9.42	67.57 ± 9.60	68.17 ± 10.57	67.14 ± 8.04	67.97 ± 9.53	0.944
Temperature ( °C)^b^, Mean ± SD	38.51 ± 0.40	38.57 ± 0.39	38.56 ± 0.45	38.45 ± 0.34	38.47 ± 0.41	0.289
NRS pain score^a^^,^^b^, Mean ± SD	7.31 ± 1.27	7.42 ± 1.22	7.20 ± 1.38	7.36 ± 1.34	7.25 ± 1.16	0.858
Gender^c^						
Male	99 (45.0%)	27 (49.0%)	30 (54.5%)	32 (58.1%)	32 (58.1%)	0.746
Female	121 (55.0%)	28 (50.9%)	25 (45.4%)	23 (41.8%)	23 (41.8%)	
Proportion of participants having fever with/without pain^c^						
Fever	220 (100.0%)	55 (100.0%)	55 (100.0%)	55 (100.0%)	55 (100.0%)	0.990
Fever with pain	169 (76.8%)	43 (78.1%)	40 (72.7%)	42 (76.3%)	44 (80.0%)	

a Analyzed for a total of 169 participants with pain.

Statistical test used:

§b One-way ANOVA.

c Chi-square test.

### 3.3 Magnitude and onset of antipyretic action

The earliest significant reduction in baseline body temperature was observed at 15 ± 2 min with fast-release test paracetamol (A), paracetamol (B), and paracetamol (C), and at 20 ± 2 min with paracetamol (D); the reduction remained significant through 90 ± 2 min for all treatments. By 90 ± 2 min, the mean body temperature decreased from 38.57 °C to 37.03 °C (Δ = 1.54 °C) with fast-release test paracetamol (A), from 38.56 °C to 37.16 °C (Δ = 1.40 °C) with paracetamol (B), from 38.46 °C to 37.29 °C (Δ = 1.17 °C) with paracetamol (C), and from 38.47 °C to 37.30 °C (Δ = 1.17 °C) with paracetamol (D) (*p* < 0.001 for all). Compared with the comparator formulations, fast-release test paracetamol (A) achieved significantly greater reductions in body temperature than paracetamol (D) from 30 ± 2 min onward (*p* < 0.05), paracetamol (C) from 45 ± 2 min onward (*p* < 0.001), and paracetamol (B) at 90 ± 2 min (*p* < 0.05) (Table 2 and Figure 2a).

**Table 2:** Body temperature (oral) from baseline till 90 ± 2 min.

	Time (min)	Fast-release test paracetamol (A) (*n* = 55)	Paracetamol (B) (*n* = 55)	Paracetamol (C) (*n* = 55)	Paracetamol (D) (*n* = 55)	Mean difference^b^ [95% CI], *p*-value for comparison of fast-release test paracetamol (A) vs.:		
						Paracetamol (B)	Paracetamol (C)	Paracetamol (D)
**Body temperature (oral; °C)**	**Baseline**	38.57 ± 0.39	38.56 ± 0.45	38.46 ± 0.34	38.47 ± 0.41	0.01 [−0.13, 0.15], 0.848	0.11 [−0.02, 0.26], 0.104	0.10 [−0.04, 0.24], 0.143
	**10 ± 2**	38.49 ± 0.41	38.48 ± 0.44	38.40 ± 0.37	38.42 ± 0.43	0.01 [−0.12, 0.16], 0.812	0.09 [−0.04, 0.24], 0.181	0.07 [−0.07, 0.21], 0.318
	**15 ± 2**	38.36 ± 0.44	38.37 ± 0.42	38.30 ± 0.38	38.33 ± 0.40	−0.01 [−0.15, 0.13], 0.941	0.06 [−0.07, 0.21], 0.358	0.03 [−0.11, 0.17], 0.649
	**20 ± 2**	38.19 ± 0.40	38.22 ± 0.41	38.16 ± 0.36	38.17 ± 0.41	−0.03 [−0.17, 0.11], 0.677	0.03 [−0.11, 0.17], 0.692	0.02 [−0.12, 0.16], 0.769
	**30 ± 2**	37.96 ± 0.42	37.97 ± 0.36	37.97 ± 0.36	37.99 ± 0.38	−0.01 [−0.16, 0.12], 0.826	−0.01 [−0.15, 0.13], 0.872	−0.03 [−0.17, 0.11], 0.664
	**45 ± 2**	37.64 ± 0.35	37.66 ± 0.32	37.75 ± 0.32	37.76 ± 0.34	−0.02 [−0.15, 0.13], 0.886	−0.11 [−0.25, 0.03], 0.140	−0.12 [−0.26, 0.02], 0.103
	**60 ± 2**	37.31 ± 0.28	37.41 ± 0.32	37.49 ± 0.29	37.51 ± 0.29	−0.10 [−0.24, 0.04], 0.180	−0.18 [−0.32, −0.04], 0.012*	−0.20 [−0.34, −0.06], 0.006**
	**90 ± 2**	37.03 ± 0.33	37.16 ± 0.38	37.29 ± 0.36	37.30 ± 0.30	−0.13 [−0.28, 0.00], 0.058	−0.26 [−0.40, −0.12], <0.001***	−0.27 [−0.41, −0.13], <0.001***
**Mean change**^a^ **from baseline [95% CI], *p*-value**	**10 ± 2**	−0.08 [−0.22, 0.06], 0.722	−0.08 [−0.22, 0.06], 0.676	−0.06 [−0.20, 0.09], 0.932	−0.05 [−0.18, 0.10], 0.983	0.00 [−0.13, 0.13], 0.960	−0.02 [−0.15, 0.11], 0.753	−0.03 [−0.16, 0.10], 0.613
	**15 ± 2**	−0.21 [−0.35, −0.07], <0.001***	−0.19 [−0.33, −0.05], 0.001**	−0.16 [−0.30, −0.02], 0.017*	−0.14 [−0.28, 0.01], 0.070	−0.02 [−0.15, 0.11], 0.774	−0.05 [−0.18, 0.08], 0.442	−0.07 [−0.20, 0.06], 0.274
	**20 ± 2**	−0.38 [−0.52, −0.24], <0.001***	−0.34 [−0.48, −0.19], <0.001***	−0.30 [−0.43, −0.15], <0.001***	−0.30 [−0.44, −0.15], <0.001***	−0.04 [−0.17, 0.09], 0.511	−0.08 [−0.22, 0.04], 0.182	−0.08 [−0.21, 0.05], 0.205
	**30 ± 2**	−0.61 [−0.76, −0.47], <0.001***	−0.59 [−0.73, −0.44], <0.001***	−0.49 [−0.63, −0.35], <0.001***	−0.48 [−0.62, −0.34], <0.001***	−0.02 [−0.16, 0.10], 0.656	−0.12 [−0.26, 0.00], 0.053	−0.13 [−0.26, −0.01], 0.040*
	**45 ± 2**	−0.93 [−1.07, −0.79], <0.001***	−0.90 [−1.04, −0.76], <0.001***	−0.71 [−0.85, −0.56], <0.001***	−0.71 [−0.85, −0.56], <0.001***	−0.03 [−0.15, 0.11], 0.717	−0.22 [−0.35, −0.09], <0.001***	−0.22 [−0.35, −0.09], <0.001***
	**60 ± 2**	−1.26 [−1.40, −1.12], <0.001***	−1.15 [−1.29, −1.01], <0.001***	−0.97 [−1.10, −0.82], <0.001***	−0.96 [−1.10, −0.82], <0.001***	−0.11 [−0.24, 0.02], 0.097	−0.29 [−0.43, −0.17], <0.001***	−0.30 [−0.43, −0.17], <0.001***
	**90 ± 2**	−1.54 [−1.69, −1.40], <0.001***	−1.40 [−1.54, −1.26], <0.001***	−1.17 [−1.31, −1.03], <0.001***	−1.17 [−1.31, −1.03], <0.001***	−0.14 [−0.28, −0.02], 0.024*	−0.37 [−0.51, −0.25], <0.001***	−0.37 [−0.50, −0.25], <0.001***

*p < 0.05; **p < 0.01; ***p < 0.001.

a Mean change = Mean (Post-baseline temperature) – Mean (baseline temperature).

b Mean difference = Mean (test medication) – Mean (reference medication) (NOTE: The between-group differences for the change from baseline does not report %diff).

Statistical test used: Repeated measures ANOVA.

Bold values indicate statistically significant *p*-values.

**Figure 2:**
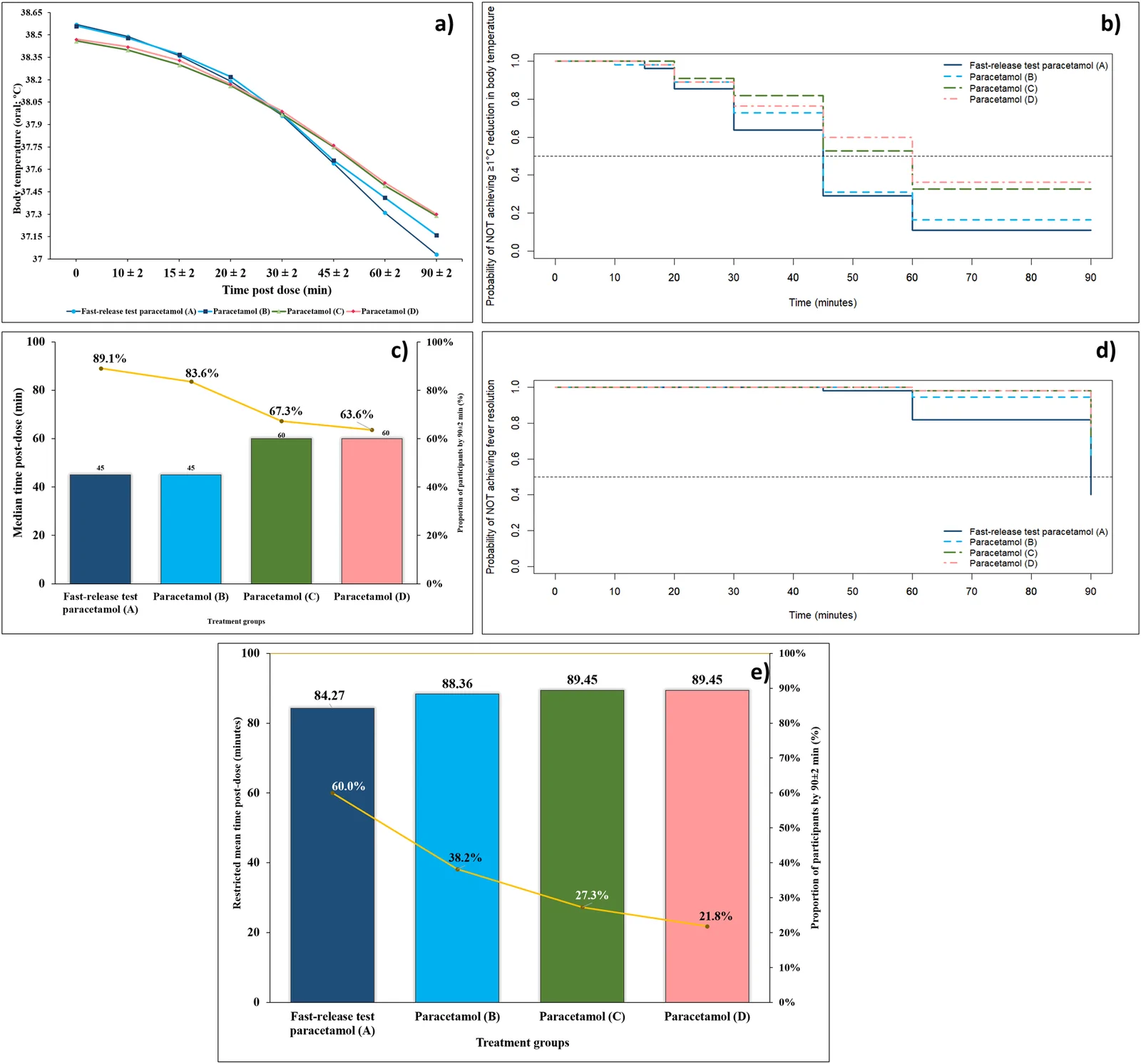
Comparison of antipyretic efficacy **(a)** body temperature from baseline through 90 ± 2 min **(b)** Kaplan–meier curves for time to ≥1 °C reduction in baseline body temperature **(c)** clinically meaningful temperature reduction: proportion of participants and median time of achievement **(d)** Kaplan–meier curves for fever resolution **(e)** defervescence: proportion of participants and restricted mean time of achievement.

The rate of temperature reduction was highest with fast-release test paracetamol (A) (1.03 °C/h), demonstrating a statistically significant advantage over paracetamol (C) and paracetamol (D) (0.7 °C/h for both; *p* < 0.001). Although the reduction rate was also higher than that of paracetamol (B) (0.9 °C/h), the difference was not statistically significant (*p* = 0.150).

For the fast-release test paracetamol (A), the decrease in the probability of not achieving the target reduction (≥1 °C reduction from baseline body temperature) indicates the accumulation of events over time. At 45 min, this probability crossed 0.5, implying that more than 50% of patients had achieved the target reduction, corresponding to the reported median time of 45 min. By 60 min, the probability declined further to approximately 0.1, indicating that nearly 90% of patients had achieved the endpoint, consistent with the observed event rate of 89.09%. Similar trends were observed for paracetamol (B), while paracetamol (C) and paracetamol (D) showed a slower decline, with median times of 60 min, reflecting a delayed onset of antipyretic action (Table 3, Figures 2b,c).

**Table 3:** Temperature reduction and fever resolution.

Characteristic	Total (*N* = 220)	Fast-release test paracetamol (A) (*n* = 55)	Paracetamol (B) (*n* = 55)	Paracetamol (C) (*n* = 55)	Paracetamol (D) (*n* = 55)
≥1 °C reduction in baseline body temperature (oral)					
*n* (%)	167 (75.9%)	49 (89.0%)	46 (83.6%)	37 (67.2%)	35 (63.6%)
*Time (min) to* ≥*1 °C reduction in baseline body temperature (oral)*^b^					
Median	45	45	45	60	60
95% CI	(45, 60)	(45, 45)	(45, 45)	(45, 60)	(45, NA^a^)
min, max	10, 90	15, 90	10, 90	20, 90	15, 90
*Comparison with Tablet A* ^c^					
Hazard ratio (HR)	NA	NA	0.85	0.51	0.47
95% CI			(0.57, 1.27)	(0.34, 0.79)	(0.30, 0.73)
*p*-value			0.418	0.002**	<0.001***
Fever resolution					
*n* (%)	81 (36.8%)	33 (60.0%)	21 (38.1%)	15 (27.2%)	12 (21.8%)
*Comparison with Tablet A (based on proportion)* ^d^					
Odds ratio (OR)	NA	NA	0.41	0.25	0.19
95% CI			[0.19, 0.88]	[0.11, 0.55]	[0.08, 0.42]
*p*-value			**0.023** *	<0.001***	<0.001***
*Time (min) to fever resolution* ^b^					
Restricted mean	87.89	84.27	88.36	89.45	89.45
95% CI	-	(90, NA^a^)	-	-	-
min, max	45, 90	45, 90	60, 90	60, 90	60, 90
*Comparison with Tablet A (based on time)* ^c^					
Hazard ratio (HR)	NA	NA	0.51	0.34	0.26
95% CI			(0.29, 0.88)	(0.18, 0.62)	(0.13, 0.51)
*p*-value			**0.016** *	<0.001***	<0.001***

*p < 0.05; **p < 0.01; ***p < 0.001.

a Number of “events” within 90 ± 2 min was not enough. So, Kaplan–Meier could not estimate the tail of the survival curve. Hence, the upper CI is NA.

**Statistical test used:**

b Kaplan–Meier Estimate.

c Cox Proportional Hazards Model (Test Tablet A considered the reference group).

d Binary logistic regression.

Bold values indicate statistically significant *p*-values.

This clinically meaningful temperature drop was observed in a higher proportion of participants treated with fast-release test paracetamol (A) (89.0%) compared to paracetamol (B) (83.6%), paracetamol (C) (67.2%), and paracetamol (D) (63.6%) (Table 3 and Figure 2c). Compared to participants treated with fast-release test paracetamol (A), those treated with paracetamol (C) had a 49% lower likelihood of achieving at least a 1 °C temperature drop within 90 ± 2 min, while those treated with paracetamol (D) had a 53% lower likelihood, both of which were statistically significant (*p* = 0.002 and *p* < 0.001, respectively) (Table 3). Overall, fast-release test paracetamol (A) and paracetamol (B) demonstrated superior antipyretic efficacy, rapidly initiating temperature reduction, with fast-release test paracetamol (A) showing a slight advantage, whereas paracetamol (C) and paracetamol (D) exhibited comparatively lower response rates, with more than one-third of participants failing to achieve a ≥1 °C reduction.

No patient achieved fever resolution before 45 min, as reflected by the flat curves across all treatment groups during the initial period. Fast-release test paracetamol (A) showed a relatively greater decline in the probability of not achieving fever resolution compared to the other treatment groups. By 90 min, the probability for fast-release test paracetamol (A) was approximately 0.4, indicating that 60% of patients had achieved fever resolution, consistent with the observed event rate. The median time to fever resolution for this group was 90 min, corresponding to the time at which the survival probability approached 0.50. In contrast, paracetamol (B), paracetamol (C), and paracetamol (D) exhibited minimal declines in probability over time, with values remaining above 0.50 throughout the observation period. Consequently, fewer than 50% of patients in these groups achieved fever resolution within 90 min, and the median time was not estimable. The restricted mean time to fever resolution further supports these findings, with a lower mean time observed for fast-release test paracetamol (A) (84.27 min) compared to paracetamol (B) (88.36 min), paracetamol (C) (89.45 min), and paracetamol (D) (89.45 min), indicating relatively faster defervescence. Overall, the Kaplan–Meier estimates are consistent with the summary statistics, highlighting a faster and higher rate of fever resolution with fast-release test paracetamol (A) compared with the other treatments (Table 3 and Figure 2d).

Furthermore, fast-release test paracetamol (A) demonstrated comparatively better clinical outcomes, with 60.0% of participants achieving complete fever resolution by 90 ± 2 min, compared with the corresponding proportions in groups treated with paracetamol (B) (38.18%), paracetamol (C) (27.2%), and paracetamol (D) (21.8%) (Table 3 and Figure 2e). Compared to participants treated with fast-release test paracetamol (A), those treated with each of the comparator paracetamol formulations had significantly lower odds of achieving complete fever resolution by 90 ± 2 min: 59% lower for paracetamol (B) (*p* = 0.02), 75% lower for paracetamol (C) (*p* < 0.001), and 81% lower for paracetamol (D) (*p* < 0.001) (Table 3).

### 3.4 Magnitude and onset of analgesic action

Fast-release test paracetamol (A), paracetamol (B), and paracetamol (C) produced a significant reduction in baseline pain scores from 15 ± 2 min onwards, whereas the reduction with paracetamol (D) became significant only from 20 ± 2 min. By 90 ± 2 min, the mean NRS pain score had decreased significantly across all treatment groups, from 7.42 to 2.19 with fast-release test paracetamol (A), 7.20–3.43 with paracetamol (B), 7.36–3.69 with paracetamol (C), and 7.25–4.14 with paracetamol (D). The magnitude of pain reduction at 90 ± 2 min was significantly greater with fast-release test paracetamol (A) (5.23 units) than with paracetamol (B), paracetamol (C), and paracetamol (D) (3.77, 3.67, and 3.11 units, respectively; *p* < 0.001 for all comparisons). Furthermore, from 30 ± 2 min onwards, pain reduction with fast-release test paracetamol (A) remained significantly greater than that achieved with each of the comparator drugs (*p* < 0.01 for all) (Table 4 and Figure 3a).

**Table 4:** NRS pain score from baseline till 90 ± 2 min.

	Time (min)	Fast-release test paracetamol (A) (*n* = 43)	Paracetamol (B) (*n* = 40)	Paracetamol (C) (*n* = 42)	Paracetamol (D) (*n* = 44)	Mean difference [95% CI], *p*-value for comparison of Tablet A vs.:		
						Paracetamol (B)	Paracetamol (C)	Paracetamol (D)
NRS pain score	**Baseline**	7.42 ± 1.22	7.20 ± 1.38	7.36 ± 1.34	7.25 ± 1.16	0.22 [−0.71, 1.15], 0.999	0.06 [−0.86, 0.98], 0.999	0.17 [−0.74, 1.08], 0.999
	**10** **±** **2**	7.09 ± 1.15	6.88 ± 1.30	7.10 ± 1.25	7.00 ± 1.12	0.21 [−0.71, 1.15], 0.999	−0.01 [−0.92, 0.92], 0.999	0.09 [−0.82, 1.00], 0.999
	**15** **±** **2**	6.47 ± 1.01	6.58 ± 1.24	6.76 ± 1.25	6.73 ± 1.23	−0.11 [−1.04, 0.82], 0.999	−0.29 [−1.22, 0.62], 0.999	−0.26 [−1.17, 0.65], 0.999
	**20** **±** **2**	5.86 ± 1.13	5.83 ± 1.68	6.12 ± 1.31	6.21 ± 1.41	0.03 [−0.90, 0.97], 0.999	−0.26 [−1.18, 0.66], 0.999	−0.35 [−1.25, 0.57], 0.999
	**30** **±** **2**	4.63 ± 1.29	5.30 ± 1.60	5.57 ± 1.47	5.73 ± 1.47	−0.67 [−1.60, 0.26], 0.339	−0.94 [−1.86, −0.02], 0.041*	−1.10 [−2.01, −0.19], 0.009**
	**45** **±** **2**	4.07 ± 1.39	4.73 ± 1.89	4.95 ± 1.72	5.14 ± 1.61	−0.66 [−1.59, 0.28], 0.378	−0.88 [−1.80, 0.04], 0.068	−1.07 [−1.98, −0.16], 0.012*
	**60** **±** **2**	3.23 ± 1.70	4.08 ± 2.16	4.26 ± 2.11	4.61 ± 1.87	−0.85 [−1.77, 0.09], 0.102	−1.03 [−1.95, −0.11], 0.020*	−1.38 [−2.29, −0.47], <0.001***
	**90** **±** **2**	2.19 ± 2.10	3.43 ± 2.52	3.69 ± 2.40	4.14 ± 2.20	−1.24 [−2.17, −0.31], 0.003**	−1.50 [−2.42, −0.58], <0.001***	−2.22 [−2.86, −1.04], <0.001***
Mean change from baseline (% change) [95% CI], *p*-value	**10** **±** **2**	−0.33 [−0.89, 0.24], 0.826	−0.32 [−0.91, 0.26], 0.926	−0.26 [−0.83, 0.31], 0.999	−0.2 [−0.80, 0.30], 0.999	−0.01 [−0.59, 0.59], 0.998	−0.07 [−0.64, 0.52], 0.830	−0.08 [−0.65, 0.50], 0.796
	**15** **±** **2**	−0.95 [−1.51, −0.39], <0.001***	−0.62 [−1.21, −0.04], 0.027*	−0.60 [−1.16, −0.03], 0.033*	−0.52 [−1.08, 0.03], 0.078	−0.33 [−0.92, 0.26], 0.273	−0.35 [−0.94, 0.22], 0.227	−0.43 [−1.00, 0.14], 0.141
	**20** **±** **2**	−1.56 [−2.12, −1.00], <0.001***	−1.37 [−1.96, −0.79], <0.001***	−1.24 [−1.81, −0.67], <0.001***	−1.04 [−1.60, −0.49], <0.001***	−0.19 [−0.77, 0.41], 0.541	−0.32 [−0.90, 0.26], 0.280	−0.52 [−1.09, 0.06], 0.080
	**30** **±** **2**	−2.79 [−3.35, −2.23], <0.001***	−1.90 [−2.48, −1.32], <0.001***	−1.79 [−2.35, −1.22], <0.001***	−1.52 [−2.08, −0.97], <0.001***	−0.89 [−1.48, −0.30], 0.003**	−1.00 [−1.59, −0.42], <0.001***	−1.27 [−1.84, −0.69], <0.001***
	**45** **±** **2**	−3.35 [−3.91, −2.79], <0.001***	−2.47 [−3.06, −1.89], <0.001***	−2.41 [−2.97, −1.84], <0.001***	−2.11 [−2.67, −1.56], <0.001***	−0.88 [−1.46, −0.29], 0.004**	−0.94 [−1.52, −0.36], <0.001***	−1.24 [−1.81, −0.66], <0.001***
	**60** **±** **2**	−4.19 [−4.75, −3.63], <0.001***	−3.12 [−3.71, −2.54], <0.001***	−3.10 [−3.66, −2.53], <0.001***	−2.64 [−3.19, −2.08], <0.001***	−1.07 [−1.65, −0.47], <0.001***	−1.09 [−1.67, −0.51], <0.001***	−1.55 [−2.12, −0.98], <0.001***
	**90** **±** **2**	−5.23 [−5.79, −4.67], <0.001***	−3.77 [−4.36, −3.19], <0.001***	−3.67 [−4.23, −3.10], <0.001***	−3.11 [−3.67, −2.56], <0.001***	−1.46 [−2.05, −0.87], <0.001***	−1.56 [−2.15, −0.98], <0.001***	−2.12 [−2.69, −1.54], <0.001***

*p < 0.05; **p < 0.01; ***p < 0.001.

a Mean change = Mean (Post-baseline NRS score)—Mean (baseline NRS score).

b Mean difference = Mean (test medication) – Mean (reference medication) (NOTE: The between-group differences in change from baseline are reported as absolute differences, not percentage differences.

Statistical tests used:

• Linear Mixed Model was used to compare the change in pain score based on the NRS scale to assess the fixed effects of treatment arm, time and interaction effect of treatment arm & time.

• EMMs and corresponding 95% CIs were derived from the fitted model for each treatment group at each time point.

• Multiplicity adjustment was applied using Bonferroni method.

Bold values indicate statistically significant *p*-values.

**Figure 3:**
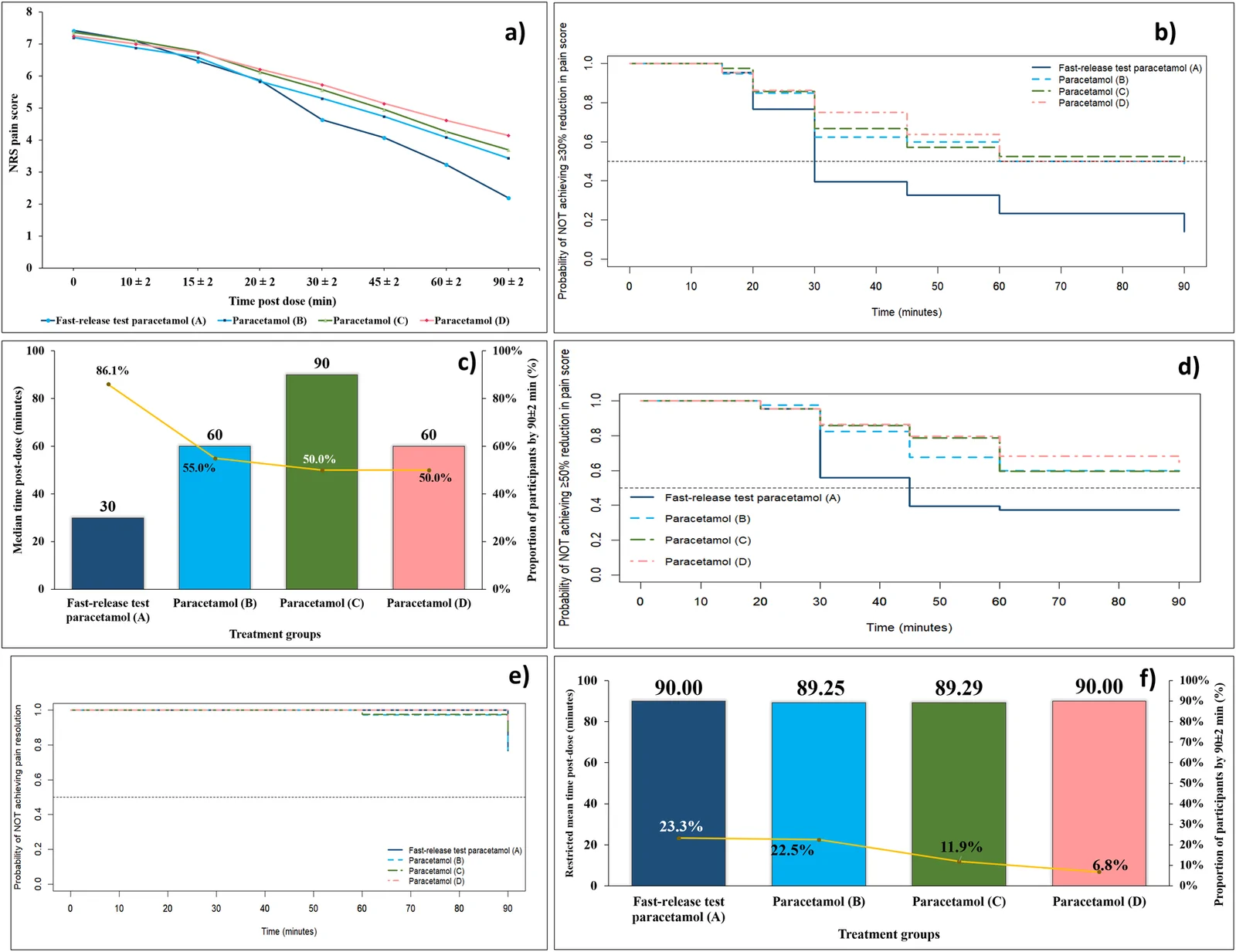
Comparison of analgesic efficacy **(a)** pain score from baseline through 90 ± 2 min **(b)** Kaplan Meier curves for time to ≥30% reduction in baseline pain score **(c)** clinically meaningful pain reduction: proportion of participants and median time of achievement **(d)** Kaplan Meier curves for time to ≥50% reduction in baseline pain score (**e)** Kaplan Meier curves for pain resolution **(f)** complete pain resolution: proportion of participants and the restricted mean time of achievement.

Fast-release test paracetamol (A) also demonstrated the fastest rate of pain reduction (3.49 units/h), which was significantly higher than that observed with paracetamol (B) (2.52 units/h; *p* = 0.011), paracetamol (C) (2.44 units/h; *p* = 0.006), and paracetamol (D) (2.08 units/h; *p* < 0.001).

Fast-release test paracetamol (A) demonstrated the most rapid onset of clinically meaningful analgesia, defined as the first perceptible ≥30% reduction in baseline NRS pain score. At 30 min, the probability of not achieving the endpoint declined to approximately 0.40, indicating that nearly 60% of participants had already achieved a clinically meaningful reduction in pain, corresponding to the median onset time of 30 min. By 90 min, this probability further decreased to approximately 0.14, consistent with an event rate of 86.0%. In comparison, paracetamol (B) and paracetamol (D) showed slower declines, with survival probabilities approaching 0.50 at 60 min, indicating median onset times of 60 min. Paracetamol (C) exhibited the slowest response, with the probability remaining above 0.50 until 90 min, corresponding to a median onset time of 90 min and an overall event rate of 50%. Overall, the Kaplan–Meier estimates were consistent with the summary statistics, with median onset times corresponding to the time at which 50% of participants achieved the endpoint. Fast-release test paracetamol (A) achieved a median onset of analgesic action of 30 min, approximately twice as fast as the comparator drugs, for which the median onset ranged from 60 to 90 min (Table 5, Figures 3b,c).

**Table 5:** Clinically meaningful pain reduction and pain resolution.

Outcome/Parameter	Total (*N* = 169)	Fast-release test paracetamol (A) (*n* = 43)	Paracetamol (B) (*n* = 40)	Paracetamol (C) (*n* = 42)	Paracetamol (D) (*n* = 44)
≥30% reduction in baseline NRS pain score					
*n* (%)	102 (60.3%)	37 (86.0%)	22 (55.0%)	21 (50.0%)	22 (50.0%)
*Time (min) to* ≥*30% reduction in baseline NRS score*^b^					
Median	60	30	60	90	60
95% CI	(45, 90)	(30, 60)	(30, NA^a^)	(45, NA^a^)	(60, NA^a^)
min, max	15, 90	15, 90	15, 90	15, 90	15, 90
*Comparison with Tablet A* ^c^					
Hazard ratio (HR)	NA	NA	0.48	0.41	0.40
95% CI			(0.28, 0.82)	(0.24, 0.71)	(0.23, 0.68)
*p*-value			0.007**	0.001**	<0.001***
Pain resolution					
*n* (%)	27 (15.9%)	10 (23.2%)	9 (22.5%)	5 (11.9%)	3 (6.8%)
*Comparison with Tablet A* ^d^					
Odds ratio (OR)	NA	NA	0.96	0.45	0.24
95% CI			[0.34, 2.69]	[0.13, 1.39]	[0.05, 0.86]
*p*-value			0.935	0.177	**0.042** *
*Time (min) to pain resolution (NRS score=0)*^b^,^d^					
Restricted mean	89.64	90.00	89.25	89.29	90.00
min, max	20, 90	90, 90	20, 90	60, 90	90, 90
*Comparison with Tablet A* ^c^					
Hazard ratio (HR)	NA	NA	1.12	0.49	0.27
95% CI			(0.46, 2.68)	(0.17, 1.43)	(0.07, 0.98)
*p*-value			0.807	0.193	**0.047** *
≥50% reduction in baseline NRS pain score					
*n* (%)	76 (45.0%)	27 (62.8%)	16 (40.0%)	17 (40.5%)	16 (36.4%)
*Time (min) to* ≥*50% reduction in baseline NRS score*					
Restricted mean	68.02	55.00	70.25	72.02	74.89
95% CI	–	(30, NA^a^)	(60, NA^a^)	(60, NA^a^)	-
min, max	20, 90	20, 90	20, 90	20, 90	20
*Comparison with Tablet A* ^c^					
Hazard ratio (HR)	NA	NA	0.47	0.45	0.40
95% CI			(0.25, 0.87)	(0.25, 0.84)	(0.21, 0.74)
*p*-value			0.017*	0.011*	0.004**

*p < 0.05; **p < 0.01; ***p < 0.001.

a Number of “events” within 90 ± 2 min was not enough. So, Kaplan–Meier could not estimate the tail of the survival curve. Hence, the upper CI is NA.

Statistical test used:

b Kaplan–Meier Estimate.

c Cox Proportional Hazards Model (Test Tablet A considered the reference group for statistical analysis);

d Binary Logistic regression.

CI could not be calculated since 50% of the patients did not achieve complete pain resolution.

Bold values indicate statistically significant *p*-values.

Although paracetamol (B) was the only comparator to demonstrate clinically meaningful pain reduction as early as 15 ± 2 min, fast-release test paracetamol (A) consistently produced the highest proportion of responders from 20 ± 2 min through 90 ± 2 min. Overall, 86.0% of participants receiving fast-release test paracetamol (A) achieved a ≥30% reduction in baseline pain score by 90 ± 2 min, compared with 55.0% for paracetamol (B), 50.0% for paracetamol (C), and 50.0% for paracetamol (D). Consequently, the overall study response rate of 60.36% was largely driven by the high responder rate observed with fast-release test paracetamol (A) (Table 5 and Figure 3c).

Compared with fast-release test paracetamol (A), treatment with each of the comparator drugs was associated with a significantly lower likelihood of achieving a ≥30% reduction in pain score within 90 min: 52% lower for paracetamol (B) (*p* = 0.007), 59% lower for paracetamol (C) (*p* = 0.001), and 60% lower for paracetamol (D) (*p* < 0.001) (Table 5).

A similar treatment pattern was observed for the more stringent cut off value of ≥50% reduction in baseline NRS pain score. By 90 ± 2 min, 62.8% of participants receiving fast-release test paracetamol (A) achieved a ≥50% pain reduction, compared with 40.0% with paracetamol (B), 40.5% with paracetamol (C), and 36.4% with paracetamol (D). The restricted mean time to achieve ≥50% pain reduction was shortest with fast-release test paracetamol (A) (55.00 min), compared with 70.25 min, 72.02 min, and 74.89 min with paracetamol (B), paracetamol (C), and paracetamol (D), respectively. Relative to fast-release test paracetamol (A), the likelihood of achieving ≥50% pain reduction was significantly lower with paracetamol (B) (HR = 0.47; 95% CI: 0.25–0.87; *p* = 0.017), paracetamol (C) (HR = 0.45; 95% CI: 0.25–0.84; *p* = 0.011), and paracetamol (D) (HR = 0.40; 95% CI: 0.21–0.74; *p* = 0.004), further supporting the faster and greater analgesic response with the fast-release formulation (Table 5 and Figure 3d).

Across all treatment groups, the Kaplan–Meier curves for complete pain resolution remained close to 1.0 throughout the observation period, indicating that most participants did not achieve complete analgesia within 90 min. Only minimal declines in the probability of not achieving complete pain resolution were observed toward the end of follow-up, and because fewer than 50% of participants reached complete pain resolution in any treatment group, the median time to event could not be estimated. The restricted mean time to complete pain resolution ranged from approximately 89–90 min across all treatment groups (Figure 3e).

By 90 ± 2 min, fast-release test paracetamol (A) and paracetamol (B) achieved the highest rates of complete pain resolution (NRS score = 0), with 23.2% and 22.5% of participants attaining complete analgesia, respectively. In contrast, complete pain resolution was achieved by only 11.9% of participants receiving paracetamol (C) and 6.8% receiving paracetamol (D). These findings, together with the substantially higher proportion of participants achieving ≥30% pain reduction with fast-release test paracetamol (A), indicate superior overall analgesic efficacy and a more rapid onset of symptom relief compared with the comparator formulations (Table 5, Figures 3d,f).

Although the restricted mean time to complete pain resolution with paracetamol (D) (90.00 min) was similar to that of fast-release test paracetamol (A), participants receiving paracetamol (D) had a significantly lower likelihood of achieving complete pain resolution within 90 ± 2 min (HR = 0.27; *p* < 0.05). The corresponding restricted mean times were 89.25 min for paracetamol (B) and 89.29 min for paracetamol (C). Participants treated with paracetamol (C) also showed a 51% lower likelihood of complete pain resolution than those receiving fast-release test paracetamol (A), although this difference was not statistically significant (HR = 0.49; *p* = 0.193). Conversely, paracetamol (B) demonstrated a 12% higher likelihood of complete pain resolution than fast-release test paracetamol (A), but this difference was not statistically significant (HR = 1.12; 95% CI: 0.46–2.68; *p* = 0.807). Overall, complete pain resolution within 90 min was achieved by a limited proportion of participants, whereas the ≥30% and ≥50% pain-reduction endpoints more clearly differentiated the faster and greater analgesic response with fast-release test paracetamol (A). (Table 5 and Figure 3e).

### 3.5 Patient-reported treatment satisfaction

High levels of patient satisfaction (“satisfied” or “very satisfied”) were reported by 82.2% of the total cohort. Satisfaction rates were highest among participants treated with fast-release test paracetamol (A) (98.1%), followed by paracetamol (B) (87.2%), paracetamol (C) (81.8%), and paracetamol (D) (61.8%) (Figure 4). Compared with fast-release test paracetamol (A), the likelihood of being satisfied was significantly lower among participants treated with paracetamol (C) (OR = 0.08; *p* = 0.020) and paracetamol (D) (OR = 0.03; *p* < 0.001).

**Figure 4:**
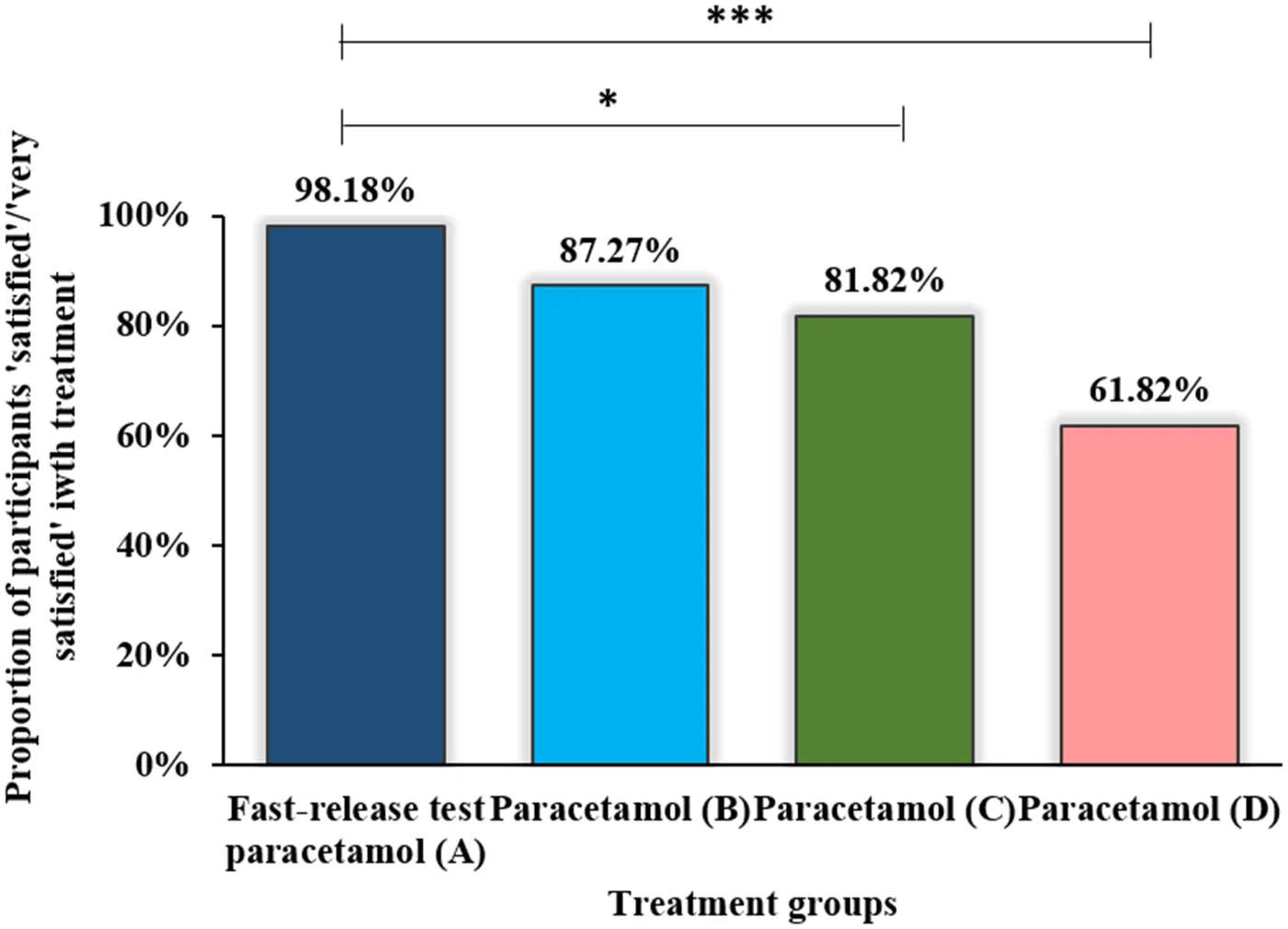
Proportion of patients with treatment satisfaction.

### 3.6 Adverse events

A total of four participants reported adverse events (AEs): one participant treated with conventional drug (C) experienced mild nausea, while three participants treated with conventional drug (D) reported mild dizziness, mild fatigue, and moderate dehydration. All AEs were assessed as unrelated to the study medication and resolved on the day of onset. No participants discontinued the study because of AEs, and no serious adverse events (SAEs) were reported.

## 4 Discussion

In this multicenter randomized controlled study, differences in the early antipyretic and analgesic responses were observed among the evaluated oral paracetamol 650 mg formulations during the initial 90-min post-dose period. The reduction in body temperature observed across all treatment groups is consistent with the well-established antipyretic activity of paracetamol. However, fast-release test paracetamol (A) demonstrated earlier reductions in both body temperature and pain intensity, with a greater proportion of participants achieving predefined clinically meaningful reductions in fever and pain within the observation period. Compared with the comparator formulations, fast-release test paracetamol (A) achieved the greatest mean reduction in body temperature (1.54 °C at 90 ± 2 min), the fastest rate of temperature reduction (1.0 °C/h), and the shortest time to clinically meaningful defervescence. Previous studies have shown that conventional 1,000 mg paracetamol produces a temperature reduction of approximately 1.0–1.1 °C at 90 min (6), indicating that formulation characteristics, rather than dose alone, may influence the onset of antipyretic response.

The clinically meaningful pain reduction occurred earlier and in a greater proportion of participants receiving fast-release test paracetamol (A) than those receiving the comparator formulations. Earlier analgesic onset is particularly relevant in acute febrile illnesses, where rapid symptom relief may improve patient comfort and treatment satisfaction. The prespecified ≥30% pain-reduction endpoint demonstrated a clear early analgesic advantage with the fast-release formulation, and this finding was further supported by the exploratory ≥50% pain-reduction analysis, which showed a consistent treatment advantage even when a more stringent threshold of pain relief was applied. Together, these findings suggest that fast-release test paracetamol provides both a faster onset and a greater depth of analgesic response. These observations are consistent with previous studies showing that rapidly acting paracetamol formulations can provide earlier analgesic relief in acute pain conditions (7, 8). Earlier pain relief has also been associated with improved patient satisfaction and more rapid functional recovery (9). Consistent with these observations, participants receiving fast-release test paracetamol (A) reported the highest levels of treatment satisfaction in the present study.

The pharmacological action of paracetamol itself is not expected to differ among marketed formulations; differences in the timing of clinical response are more plausibly related to pharmaceutical characteristics influencing drug release and absorption. Following oral administration, tablet disintegration and dissolution precede intestinal absorption, and formulation-dependent acceleration of these processes may increase the rate at which paracetamol becomes available for absorption (8). Evidence from rapidly absorbed oral analgesic formulations supports a relationship between faster absorption and earlier analgesic effect. Moore et al. demonstrated substantial formulation-dependent differences in ibuprofen absorption, with rapidly absorbed formulations achieving earlier peak plasma concentrations and faster analgesic responses than standard formulations (10). Similarly, contemporary studies of fast-acting acetaminophen have demonstrated an earlier onset of pain relief compared with conventional acetaminophen formulations (3, 11–12).

The earlier clinical response observed with fast-release test paracetamol (A) is consistent with the general pharmacological rationale that more rapidly disintegrating or dissolving formulations may provide earlier drug availability for absorption. However, pharmacokinetic assessments were not performed in the present study, and plasma paracetamol concentrations were not measured. The observed differences in antipyretic and analgesic onset cannot be directly attributed to differences in absorption rate or systemic drug exposure, and the proposed formulation-related mechanism should be regarded as biologically plausible rather than demonstrated by the present study. All evaluated formulations were generally well tolerated during the study. Reported adverse events were mild or moderate in severity, resolved without sequelae, and none were considered related to the study medication. No serious adverse events or hepatic safety concerns were observed, findings that are consistent with the well-established safety profile of single therapeutic doses of paracetamol (13). Nevertheless, the relatively short duration of follow-up precluded assessment of delayed adverse events or safety associated with repeated dosing.

The external validity of these findings is strengthened by the multicenter study design conducted across India, reflecting routine clinical practice. Although most participants presented with low-grade fever, baseline pain intensity was sufficiently high to permit meaningful evaluation of analgesic efficacy. The active-controlled design enabled direct comparison among four commonly prescribed oral paracetamol formulations available in clinical practice. Randomization resulted in balanced baseline characteristics across treatment groups, minimizing selection bias. Furthermore, standardized temperature measurements and frequent assessments allowed detailed characterization of the early time course of both antipyretic and analgesic responses. The inclusion of multiple clinically relevant efficacy endpoints and an adequately powered sample further strengthen the robustness of the findings.

Several limitations should be considered when interpreting these results. First, the open-label design may have introduced performance and reporting bias, particularly for subjective outcomes such as pain intensity and treatment satisfaction. Participants' awareness of the assigned marketed formulation and any pre-existing perceptions regarding these products may also have influenced patient-reported assessments; therefore, between-group differences in these outcomes should be interpreted with appropriate caution. In addition, the timing of study-drug administration relative to food intake was not standardized or systematically recorded, which may have contributed to variability in the early clinical response. Blinding was not feasible because of differences in tablet appearance and packaging among the marketed products. Second, follow-up was limited to 90 min; consequently, only the early onset of antipyretic and analgesic activity could be evaluated. Longer-term efficacy, duration of symptom relief, recurrence of fever or pain, repeat dosing requirements, rescue medication use, and longer-term safety were not assessed. Third, pharmacokinetic analyses were beyond the scope of the present study; the observed differences in clinical response could not be directly correlated with systemic drug exposure or absorption profiles. Finally, the underlying etiologies of fever and associated pain were heterogeneous and were not stratified, which may have contributed to variability in treatment response despite balanced randomization.

Future studies should employ blinded or double-dummy designs where feasible to minimize expectation and reporting bias, particularly for subjective outcomes such as pain intensity and treatment satisfaction. Pharmacokinetic sampling would help determine whether differences in the onset of clinical response correspond to differences in early drug absorption and systemic exposure. Future trials should also incorporate validated patient-reported outcome measures and longer follow-up, together with clinically meaningful patient-centered endpoints such as rescue medication use, duration of symptom relief, time to complete symptom resolution, recurrence of fever or pain, functional recovery, and time to return to usual daily activities. Such assessments would help determine whether differences observed during the early post-dose period translate into sustained and clinically meaningful benefits.

## 5 Conclusion

In adults with acute febrile or febrile-pain conditions, all evaluated oral paracetamol 650 mg formulations showed reductions in body temperature and pain during the 90-min observation period. The fast-release formulation showed earlier and, for several assessed outcomes, greater reductions in body temperature and pain than the conventional formulations. These findings may inform consideration of formulation choice when rapid early symptomatic relief is desired; however, they do not establish superiority in longer-term clinical outcomes. Further blinded studies incorporating pharmacokinetic assessment and longer follow-up are warranted to confirm the clinical relevance and persistence of these early differences.

## Data Availability

The original contributions presented in the study are included in the article/Supplementary Material, further inquiries can be directed to the corresponding author/s.
